# p21^WAF1 ^expression induced by MEK/ERK pathway activation or inhibition correlates with growth arrest, myogenic differentiation and onco-phenotype reversal in rhabdomyosarcoma cells

**DOI:** 10.1186/1476-4598-4-41

**Published:** 2005-12-13

**Authors:** Carmela Ciccarelli, Francesco Marampon, Arianna Scoglio, Annunziata Mauro, Cristina Giacinti, Paola De Cesaris, Bianca M Zani

**Affiliations:** 1 Department of Experimental Medicine, University of L'Aquila, L'Aquila, Italy; 2 Department of Histology and general Embryology, University of Rome "La Sapienza", Rome, Italy

## Abstract

**Background:**

p21^WAF1^, implicated in the cell cycle control of both normal and malignant cells, can be induced by p53-dependent and independent mechanisms. In some cells, MEKs/ERKs regulate p21^WAF1 ^transcriptionally, while in others they also affect the post-transcriptional processes. In myogenic differentiation, p21^WAF1 ^expression is also controlled by the myogenic transcription factor MyoD. We have previously demonstrated that the embryonal rhabdomyosarcoma cell line undergoes growth arrest and myogenic differentiation following treatments with TPA and the MEK inhibitor U0126, which respectively activate and inhibit the ERK pathway.

In this paper we attempt to clarify the mechanism of ERK-mediated and ERK-independent growth arrest and myogenic differentiation of embryonal and alveolar rhabdomyosarcoma cell lines, particularly as regards the expression of the cell cycle inhibitor p21^WAF1^.

**Results:**

p21^WAF1 ^expression and growth arrest are induced in both embryonal (RD) and alveolar (RH30) rhabdomyosarcoma cell lines following TPA or MEK/ERK inhibitor (U0126) treatments, whereas myogenic differentiation is induced in RD cells alone. Furthermore, the TPA-mediated post-transcriptional mechanism of p21^WAF1^-enhanced expression in RD cells is due to activation of the MEK/ERK pathway, as shown by transfections with constitutively active MEK1 or MEK2, which induces p21^WAF1 ^expression, and with ERK1 and ERK2 siRNA, which prevents p21^WAF1 ^expression. By contrast, U0126-mediated p21^WAF1 ^expression is controlled transcriptionally by the p38 pathway. Similarly, myogenin and MyoD expression is induced both by U0126 and TPA and is prevented by p38 inhibition. Although MyoD and myogenin depletion by siRNA prevents U0126-mediated p21^WAF1^ expression, the over-expression of these two transcription factors is insufficient to induce p21^WAF1^. These data suggest that the transcriptional mechanism of p21^WAF1 ^expression in RD cells is rescued when MEK/ERK inhibition relieves the functions of myogenic transcription factors. Notably, the forced expression of p21^WAF1 ^in RD cells causes growth arrest and the reversion of anchorage-independent growth.

**Conclusion:**

Our data provide evidence of the key role played by the MEK/ERK pathway in the growth arrest of Rhabdomyosarcoma cells. The results of this study suggest that the targeting of MEK/ERKs to rescue p21^WAF1 ^expression and myogenic transcription factor functions leads to the reversal of the Rhabdomyosarcoma phenotype.

## Background

Permanent withdrawal from the cell cycle is a crucial event during terminal differentiation. Dysfunction of either cell cycle control or differentiation machinery is responsible for deregulated growth and transformed phenotype [1]. Control of G1/S transition is regulated by a set of specific CDK and cyclin complexes, sequentially expressed, activated and degraded to ensure both entry and progress in the cell cycle [2]. In large part, the cyclin/CDK complexes are needed to phosphorylate pRb, which in turn releases E2F and leads to the transcription of growth regulating genes such as cyclin A [3].

p21^WAF1^, a cyclin-dependent kinase inhibitor (CKI), which inhibits all cyclin/CDK complexes, particularly those in the G1 phase, has been found to be associated with the growth arrest of both normal and malignant cells [4]. Enhanced p21^WAF1 ^mRNA expression occurs through both p53-dependent and -independent mechanisms [5,6], and as a result of mRNA and protein stabilization induced in a number of different cell lines and signal transduction mechanisms [6-9].

In myogenic cells, muscle-specific transcription factors, such as MyoD, induce transcription of p21^WAF1 ^during differentiation [10,11], while in mice lacking MyoD and myogenin, muscle precursors correctly express p21^WAF1^, suggesting that this important cell cycle molecule is controlled by a redundant transcription factor regulatory mechanism [12]. Although hypo-phosphorylated pRb expression is up regulated during myoblast-to-myotube transition and after myogenic differentiation, the pRb kinases CDK4 and CDK6 are constitutively expressed, while CDK2 undergoes down-regulation during terminal myogenic differentiation [10,11].

The MEK/ERK pathways control the growth and survival of a broad spectrum of human tumors [13], and have also been involved in differentiation [14-16]. Indeed, a role of the MEK/ERK pathway in growth inhibition has been reported to be dependent upon whether activation is acute or chronic [17]. Although ERKs are constitutively activated in tumor growth and are involved in the induction of proliferation, a high p38 level is believed to be a negative regulator [18,19]. Furthermore, the ERK and p38 pathways have recently been reported to cooperate to cause sustained G1 cell cycle arrest requiring p21^WAF1 ^expression [20].

Rhabdomyosarcoma (RMS), the most common soft-tissue sarcoma arising from undifferentiated mesenchymal cells bearing developing skeletal muscle features, consists of several subtypes, with ERMS, the embryonal subtype, and ARMS, the alveolar subtype, being among the most frequent tumors in children [21]. RMS presents a number of genetic alterations which define the embryonal [22,23] and the alveolar subtype [24]. These different subtypes also share molecular changes, including disruption of the p53 pathway through mutation or MDM2 amplification, and deregulation of imprinted genes at the chromosome region 11p15.5 [22,25].

The established RD cell line, originating from the ERMS tumor, is one of the most representative models of pathological myogenesis. RD cells fail to control cell cycle mechanisms [26] and differentiation progress in spite of the expression of the myogenic-specific transcription factors MyoD and myogenin, which are transcriptionally inactive despite apparently being able to bind DNA [23,27]. MyoD and myogenin, when ectopically expressed in RD cells, do not induce muscle differentiation, even in the presence of cyclin-dependent kinase inhibitors (CKIs) or myogenic co-factors [28], while ectopic expression of MRF4, which is undetectable in RD, induces exit from the cell cycle and myogenic differentiation, both of which are enhanced in the presence of CKIs [29].

In a recent paper, we demonstrated that PKC-α-mediated MAPK (ERKs, JNKs and p38) activation is responsible for orchestrating growth arrest and myogenic differentiation induced by the phorbol ester TPA [30]. It is noteworthy that the use of the specific MEK inhibitor allowed us to selectively inhibit MAPK activation, thereby showing that ERKs represent the key pathway to growth arrest and myogenic differentiation when either activated or inhibited.

In this paper we attempt to clarify the mechanism of ERK-mediated and ERK-independent growth arrest and myogenic differentiation in RD cells, particularly with regard to the expression of proteins involved in cell cycle control, such as p21^WAF1^. We demonstrate that p21^WAF1 ^expression is post-transcriptionally regulated by TPA-mediated MEK/ERK activation, but transcriptionally induced by MEK/ERK inhibition and p38 activation.

Furthermore, we present evidence of p21^WAF1^ expression-dependence on myogenin and MyoD activity. In spite of the features shared by growth arrest and p21^WAF1 ^enhanced expression, RH30 cells do not undergo myogenic differentiation either under TPA or MEK inhibitor treatments. In this paper we also show that p21^WAF1 ^is involved in regulating anchorage-independent growth of RD cells.

## Results

### Sustained post-transcriptional and transient transcriptional p21^WAF1 ^expression respectively after ERK pathway activation and down-regulation

In order to identify the molecular mechanism of G1 arrest following ERK activation and MEK/ERK inhibition in RD cells (see additional file 1), we first determined the pattern of G1/S cyclins, CDKs and CDK inhibitor proteins after TPA and U0126 treatments.

**Figure 1:**
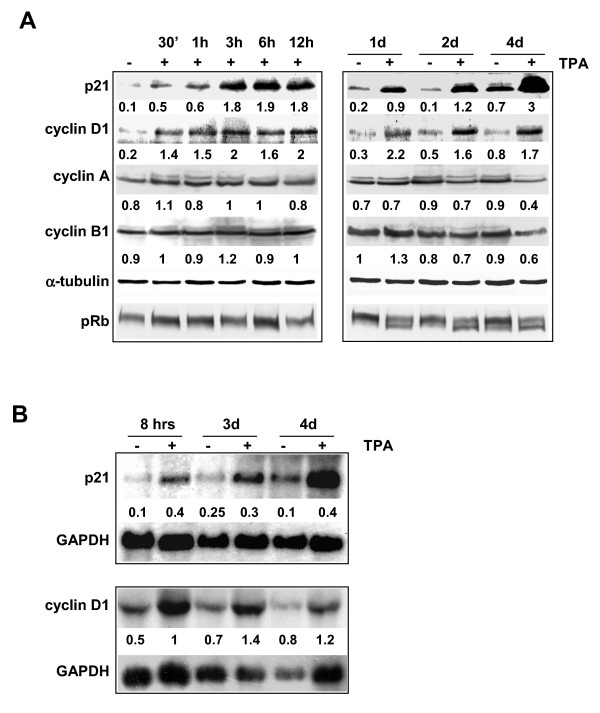
**Expression of cell cycle markers during growth arrest induced by TPA. **RD cells were treated with 10^-7 ^M TPA for the times indicated. (A) whole cell lysates from untreated (-) or TPA-treated cells (+) were separated on 12% SDS-PAGE and analysed by immunoblotting with specific antibodies for the proteins indicated. α-tubulin expression shows equal loading. pRb was analysed on a filter from 7% SDS-PAGE. Densitometric analysis of bands provided quantification expressed as the ratio of cell cycle protein amount versus the α-tubulin amount. (B) Northern blots from total RNA of untreated (-) and TPA-treated cells (+). The loading was tested by reprobing the same filters with GAPDH. Densitometric analysis of bands provided quantification as the ratio of the amount of mRNA cell cycle protein versus mRNA GAPDH amount. The data shown are representative of three independent experiments.

**Figure 2:**
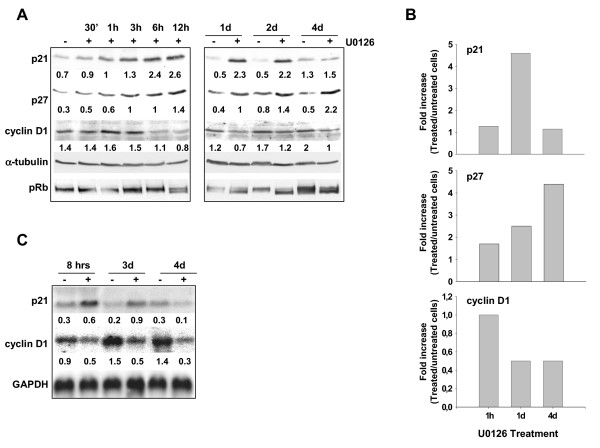
**Expression of cell cycle markers modulated by U0126.**RD cells were treated with 10 μM U0126 for the times indicated. (A) whole cell lysates from untreated (-) or U0126-treated cells (+) were separated on 12% SDS-PAGE and analysed by immunoblotting with specific antibodies for the proteins indicated. α-tubulin expression shows equal loading. pRb was analysed on a filter from 7% SDS-PAGE. Densitometric analysis of bands provided quantification expressed as the ratio of the amount of cell cycle protein versus α-tubulin amount. (B) Graph shows the quantification of the results presented in A expressed as a fold increase of the indicated cell cycle protein in the U0126 sample over the untreated sample at the time points examined. (C) Northern blot from total RNA of untreated (-) and U0126-treated cells (+). Loading was tested by reprobing the same filter with GAPDH. Densitometric analysis of bands provided quantification as the ratio of the amount of mRNA cell cycle protein versus mRNA GAPDH amount. The data shown are representative of two independent experiments.

Total lysates from RD cells, left untreated or treated with TPA for different time intervals, were analysed by immunoblotting with a panel of antibodies aimed at the cell cycle proteins. We analysed the expression level of p21^WAF1^, known to particularly inhibit the G1 cell cycle complexes. p21^WAF1 ^was rapidly (after 30 min), markedly (5–18 fold from 30 min to 12 hrs) and permanently (4 fold up to 4 days) increased by TPA treatment (Fig. 1A). Remarkably, the p21^WAF1 ^level in the control cells remained steady up to 2 days and increased at 4 days, even though TPA-induced p21^WAF1 ^expression exceeded by far the level of its respective control. Moreover, cyclin D1 was up-regulated early and permanently during TPA treatment (Fig. 1A, 30 min-4 days), while cyclin A and B1 were down-regulated later (2–4 days). p27, CDK2, CDK4, cyclin D3 and cyclin E were virtually unaltered throughout the treatment (see additional file 2). To corroborate the G1 arrest pattern, we investigated the pRb phosphorylation level, known to be down-regulated during G1 arrest [31]. pRb was heavily phosphorylated in both untreated and TPA-treated cells during the first 12 hours of treatment; by contrast, from after 1 day up to 4 days of treatment the hypo-phosphorylated isoform was easily detectable (Fig. 1A), suggesting that inhibition of G1/S progression occurs. Northern blot experiments revealed a sustained increase in p21^WAF1 ^and cyclin D1 mRNAs (Fig. 1B) after both early and prolonged TPA treatment. We then analysed p21^WAF ^and cyclin D1 expression by immunoblotting total lysates from RD cells, left untreated or treated with the MEK inhibitor U0126. Similarly to TPA, U0126 induced an early increase in p21^WAF1 ^(30 min-2 days), though at varying levels (1.2–3.7 fold from 30 min up to 12 hrs, and 4.4 fold at 2 days) (Fig. 2A). While p21^WAF1^ expression was sustained following TPA treatment (Fig. 1, 30 min-4 days), in U0126-treated cells it decreased from 2 to 4 days after treatment, as shown in Figure 2A. Notably, p27 expression progressively increased in U0126-treated cells from 1.7 (30 min) to 4.4 fold if compared with control untreated cells. Unlike p21^WAF1^, cyclin D1 expression dropped to below the level of control untreated cells from as early as 6 hours and up to 4 days after treatment. In addition, the hypo-phosphorylated form of pRb was detected as early as 12 hours after the start of treatment and was sustained for up to 4 days. Interestingly, the quantification of the levels of p21^WAF1^, p27 and cyclin D1 expression in early (1 hr), middle (1 day) and late (4 day) U0126 treatments shows how key cell cycle protein levels are inversely correlated, with p21^WAF1 ^dropping when p27 peaks and cyclin D1 also drops (Fig. 2B). Northern blot analysis shows that the p21^WAF1 ^transcript increases during the first day of U0126 treatment, before dropping to the basal level after prolonged treatment (from 3 to 4 days), whereas the cyclin D1 transcript is down-regulated by the inhibitor, thereby confirming the protein pattern in Western blot (Fig. 2C).

**Figure 3:**
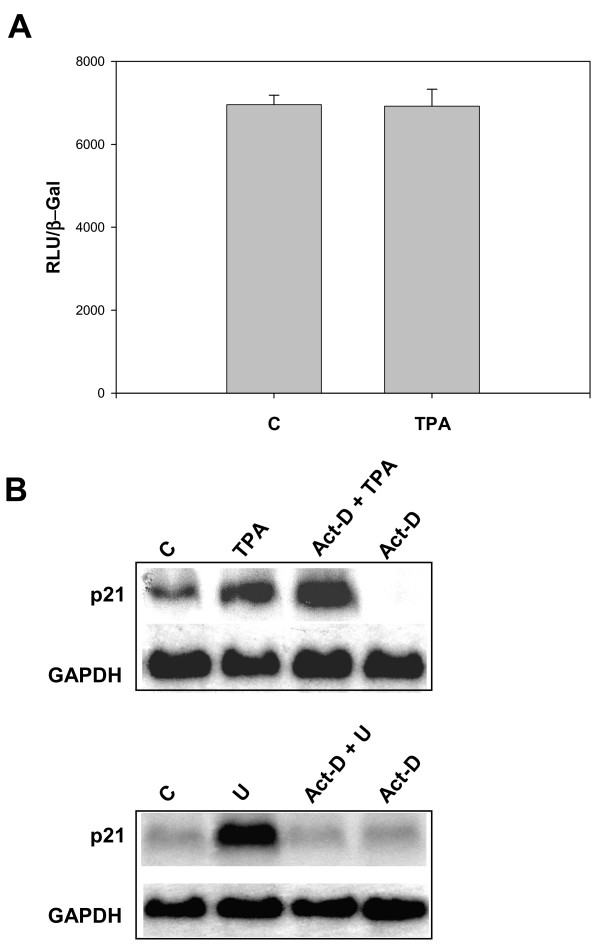
**mRNA stabilization by TPA and transcriptional activation by U0126 of p21^WAF1^ expression.**(A). RD cells were transfected with plasmid expressing β-galactosidase (β-gal) gene and a plasmid carrying p21 promoter (DM-Luc). Luciferase activity was normalized for the expression levels of transfected β-gal protein. Data show mean values ± s.e.m. of triplicates of a representative experiment. (B) Northern blots from RD cells left untreated (C) or treated with TPA *(upper panel) *or U0126 (U) *(lower panel) *for 5 hours, pre-treated with 0.05 μg/ml of actinomycin D for 1 hour and then left untreated (ActD) or treated with TPA (ActD+TPA) or U0126 (ActD+U) for 5 hours. The levels of GAPDH mRNA are shown. Similar results were obtained in three independent experiments for A and two for B.

We hypothesized that the differences in the expression and accumulation of p21^WAF1 ^in cells bearing the activated (TPA-treated) or inhibited (U0126-treated) MEK/ERK pathway might be due to transcriptional and/or post-transcriptional mechanisms [7,32]. For this purpose, we first investigated ectopic p21^WAF1 ^promoter transactivation upon TPA treatment in transiently transfected cells. RD cells were transfected with a vector expressing luciferase under the control of the p21^WAF1 ^promoter (DM-Luc) together with the β-galactosidase expression vector, and were left untreated or were treated with TPA for 24 hours. Luciferase and β-galactosidase activities were evaluated in total lysates. TPA did not increase luciferase activity (Fig. 3A).

In order to ascertain whether the increase in p21^WAF1 ^mRNA was a result of mRNA stabilization, actinomycin D-pre-treated (1 hr) cells were left untreated or were treated with TPA for 5 hours, and mRNAs were analysed in Northern blot, as shown in Figure 3B (upper panel). In TPA-treated cells, actinomycin-D did not, unlike control untreated cells (Act-D), suppress the p21^WAF1 ^mRNA transcript (Act-D TPA). This result indicates that the TPA-mediated p21^WAF1 ^increase is a result of a post-transcriptional mechanism, which suggests mRNA stabilization [6-9].

Unlike TPA, MEK/ERK inhibition induces p21^WAF1 ^expression through a transcriptional mechanism, as demonstrated by Northern blot of U0126-treated cells after actinomycin D pre-treatment (Fig. 3B, lower panel). Pre-treatment with actinomycin D completely prevented U0126-mediated induction of the p21^WAF1 ^transcript, thereby indicating that MEK/ERK inhibition restores the p21^WAF1 ^transcription mechanism.

Furthermore, actinomycin D did not alter p21^WAF1 ^expression at the protein level in either untreated cells or TPA-treated cells, but it drastically prevented the U0126-mediated increase in the p21^WAF1 ^protein (see additional file 3). A protein stabilization mechanism was tested in cells treated with TPA for 1 hour followed by cycloheximide for varying time intervals. In TPA-treated cells, cycloheximide prevented the increase in the level of p21^WAF1^, thereby demonstrating that TPA does not induce any protein stabilization mechanism (see additional file 3 ). These data, taken as a whole, demonstrate that p21^WAF1 ^accumulation is a result of post-transcriptional or transcriptional mechanisms when the MEK/ERK pathway is, respectively, active or inactive, which suggests that p21^WAF1^-induced expression is an early event in the attainment of growth arrest that is targeted by opposite pathways.

### Sustained post-transcriptional p21^WAF1 ^expression is dependent on ERK activation

**Figure 4:**
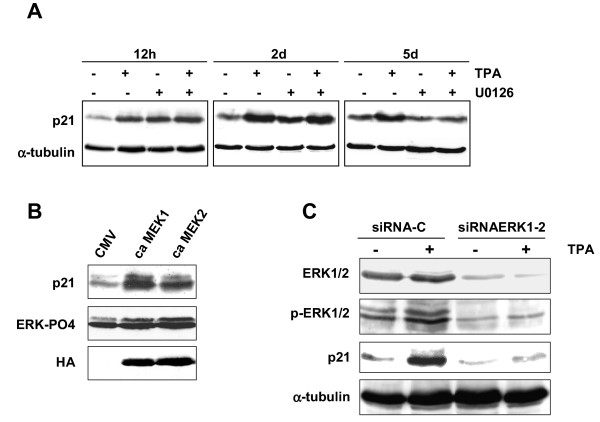
**MEK/ERK pathway sustains p21 ^WAF1 ^expression. **(A) RD cells were left untreated (-) or treated with TPA in the absence or in the presence of U0126 for the times indicated. (B) RD cells were transfected with the constitutively active form of HA-tagged-MEK1 (ca MEK1), -MEK2 (ca MEK2) or with the empty vector (CMV). (C) RD cells were transfected with control siRNA (siRNA-C) or ERK1 and ERK2 siRNAs (siRNAERK1-2) and then left untreated (-) or treated with TPA (+) for 4 days. Immunoblots of total lysates were performed using specific antibodies capable of recognising p21^WAF1^, total and phosphorylated ERK1/2, hemagglutinin (HA) and α-tubulin. The data shown are representative of three independent experiments for A and two for B and C.

In order to establish whether sustained activation of the MEK/ERK pathway plays a role in post-transcriptional-mediated p21^WAF1 ^accumulation, RD cells, pre-treated with U0126, were treated with TPA for different time intervals. p21^WAF1 ^protein accumulation was insensitive to MEK inhibitor treatment for up to 2 days, after which it dropped to the level of untreated control cells, paralleling U0126 treatment alone (Figure 4A). These results suggest that the post-transcriptional mechanism of p21^WAF1 ^induction might be strictly dependent on activated MEK/ERK pathway. Constitutively active MEK1 or MEK2 (Fig. 4B) and ERK1 and ERK2 siRNA (Fig. 4C) transient transfection experiments demonstrated that activated MEKs/ERKs are upstream pathways of p21^WAF1 ^expression. The forced expression of MEK1 or MEK2 induced both ERK phosphorylation and p21^WAF1^-increased expression (Fig. 4B). RNA interference experiments were performed with both ERK1 and ERK2 siRNA in order to prevent TPA-induced ERK1/2 activation. After 4 days of TPA treatment, we observed a lack of p21^WAF1 ^expression combined with the down-regulation of total and phospho-ERKs in ERK1 and ERK2 siRNA co-transfected cells, while ERK activation and p21^WAF1 ^expression were present in control transfected cells (siRNA-C) (Fig. 4C). All these experiments demonstrate that the post-transcriptional mechanism of p21^WAF1 ^expression is dependent on the ERK pathway in RD cells.

### Dependence of p21^WAF1 ^transcriptional expression and myogenic differentiation on the p38 pathway

**Figure 5:**
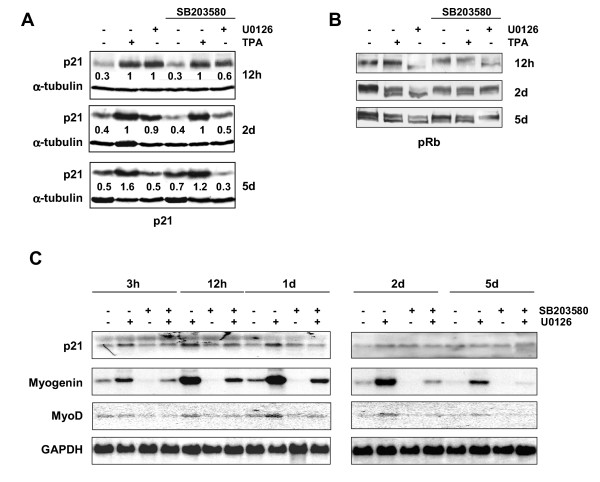
**Effects of p38 inhibition on p21^WAF1^, myogenin and MyoD expression. **(A and B) RD cells were left untreated, treated with TPA or U0126 or pre-treated with 5 μM SB 203580 for 1 hour and then left untreated or treated with TPA or U0126 for the times indicated. Immunoblots of total lysates were performed using specific antibodies recognising p21^WAF1 ^and α-tubulin (A) and pRb (B). Densitometric analysis of bands provided quantification expressed as the ratio of amount of p21^WAF1 ^versus α-tubulin amount. (C) Northern blot analysis of p21^WAF1^, myogenin and MyoD expression in RD cells left untreated or treated with U0126 in the absence or in the presence of SB 203580 for the times indicated. The GAPDH mRNA levels are shown. The data shown are representative of three independent experiments for A and B and two for C.

We have previously shown that the p38 pathway is activated concomitantly in both the activation and down-regulation of the ERK pathway, and that its inhibition prevents myogenic differentiation and reverts the growth arrest state after prolonged treatment [30]. Therefore, we investigated whether ERKs and p38 cooperate, as has recently been demonstrated in CC139 and Rat-1 cells [20], or act separately to induce p21^WAF1 ^and growth arrest. For this purpose, RD cells were treated with TPA and U0126 in the presence or absence of the p38 specific inhibitor SB203580. SB203580 treatment did not alter TPA-induced p21^WAF1 ^expression, but it did reduce U0126-induced p21^WAF1 ^expression, particularly after prolonged treatments (from 2 to 5 days, Fig. 5A); alone it only slightly enhanced the p21^WAF1 ^basal level (1.4 fold). Furthermore, p38 inhibition affected neither the TPA-induced expression of cyclin D1 nor the decrease mediated by MEK/ERK inhibition (see additional file 4). The pRb phosphorylation status is, moreover, modulated by SB203580 after 2 days in U0126 treated cells (Fig 5B). Conversely, pRb phosphorylation in TPA-treated cells is insensitive to SB203580. SB203580 alone does not affect pRb phosphorylation and the slight increase in p21^WAF1 ^expression (1.4 fold) at prolonged treatments is not sufficient to induce hypo-phosphorylation of pRb (Fig. 5B). Analysis of p21^WAF1 ^mRNA accumulation following U0126 treatment in the presence and absence of SB203580 (Fig. 5C) shows that the p21^WAF1 ^transcript was reduced by p38 inhibitor, thereby paralleling the Western blot results and suggesting that the p38 pathway is involved in transcriptional p21^WAF1 ^induction by U0126.

**Figure 6:**
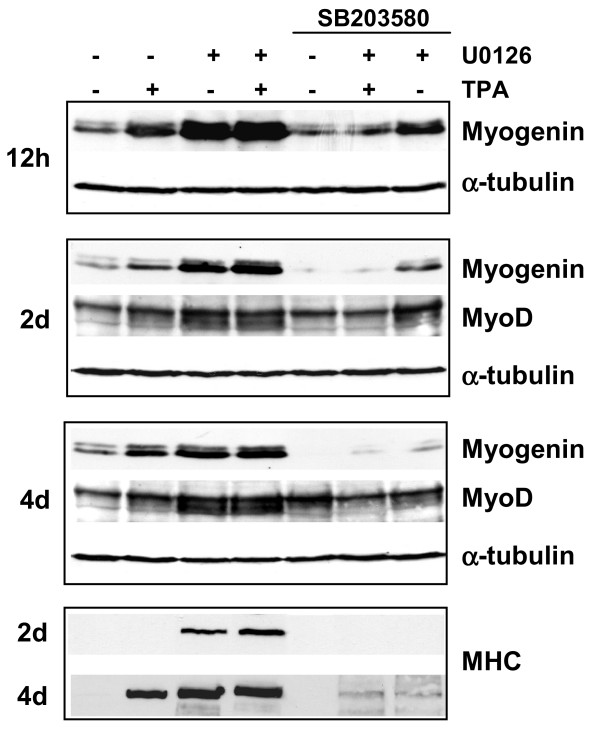
**Effects of MEK/ERKs and p38 pathways on myogenic transcription factors and myosin expression. **RD cells were left untreated, treated with TPA, treated with SB 203580 in the absence or in the presence of TPA and treated with U0126 in the absence or in the presence of TPA for the times indicated. Pre-treatment with SB 203580 or U0126 was performed for 1 hour. Immunoblots of total lysates were performed using specific antibodies recognising myogenin, MyoD and sarcomeric myosin heavy chain (MHC). α-tubulin expression shows equal loading. The data shown are representative of two independent experiments.

Since growth arrest and myogenic differentiation in ERK pathway-depleted cells is induced rapidly [30], it is possible that p21^WAF1 ^is an early downstream target of activated myogenic transcription factors, as occurs in normal myogenic myoblasts [10]. In order to verify this hypothesis, we first analysed the levels of MyoD and myogenin following U0126 treatment. The myogenin transcript was strongly enhanced in U0126-treated cells for the first day of treatment but decreased thereafter, thus resembling the pattern of the p21^WAF1 ^transcript (Fig. 5C). The increase in the MyoD transcript was also detectable from 1 day but decreased thereafter. It is noteworthy that SB203580 inhibits both myogenin and MyoD transcript expression in control untreated and in U0126-treated cells, thereby resembling the p21^WAF1 ^expression pattern. Immunoblotting analysis showed that the myogenin protein level was strongly enhanced by U0126, and to a higher degree than it was by TPA (Fig. 6). The MyoD protein level in U0126-treated cells increases together with a slow migrating form, which may be its hypo-phosphorylated isoform (Fig. 6, 2–4 days). The early induction of a differentiative pathway is corroborated by early myosin expression in 2-day U0126-treated cells, though not in the 2-day TPA-treated cells (Fig. 6). It is noteworthy that concomitant U0126 and TPA treatments of both myogenin and myosin expression are cumulative (Fig. 6). Furthermore, p38 inhibition by SB203580 reduces the slow migrating form of MyoD, as well as early and late myogenin and myosin expression in both TPA- and U0126-treated cells (Fig. 6). Lastly, to validate the efficiency of SB203580 at longer incubation times, we compared its effects on myogenin expression with those of its inactive analogue SB202474, which has been shown not to block the p38 pathway [33,34].

Treatment with SB202474 does not affect either basal or TPA-induced myogenin expression after a short or longer pre-incubation period (see additional file 5). These results demonstrate that p21^WAF1^ expression is dependent on the p38 pathway in the absence of active MEKs/ERKs, but is fully independent in the presence of activated ERKs, thereby suggesting that ERK and p38 do not cooperate in p21^WAF1^ expression.

### p21^WAF1 ^expression is dependent on MyoD and myogenin

**Figure 7:**
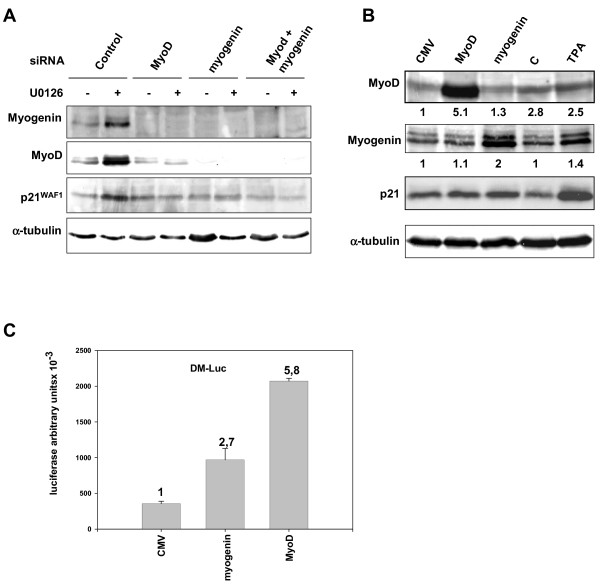
**p21^WAF1 ^expression induced by U0126 is dependent on MyoD and/or myogenin. **(A) Immunoblots of total lysates from RD cells transiently transfected with control-, MyoD- or myogenin-siRNA, or with a combination of MyoD- and myogenin-siRNA (MyoD+myogenin), and then left untreated (-) or treated with U0126 (+) for 2 days. (B) Immunoblots of total lysates from Puromycin-selected polyclonal population from RD cells transfected with the empty vector (CMV), MyoD- or myogenin-expressing vector. As a control, cells were left untransfected in the absence (C) or in the presence of TPA (TPA). Immunoblots were performed using specific antibodies capable of recognising MyoD, myogenin, p21^WAF1^ and α-tubulin. Densitometric analysis of bands provided quantification of MyoD and myogenin levels expressed as a fold increase over the control value (CMV) arbitrarily set at 1. (C) Luciferase assay of lysates from RD cells co-transfected with the empty vector (CMV), myogenin- or MyoD-expressing vector and the plasmid carrying p21^WAF1 ^promoter (DM-Luc). Data show mean values ± s.e.m. of triplicates of a representative experiment. Similar results were obtained in two experiments.

We then decided to investigate whether the transcriptional mechanism of U0126-mediated p21^WAF1 ^expression is a result of restored myogenic transcription factor function. For this purpose, we performed two different experiments designed to clarify, on the one hand, whether myogenin and MyoD depletion impairs U0126-mediated p21^WAF1 ^expression and, on the other, whether their increased levels rescue p21^WAF1 ^expression. We first performed siRNA experiments using control, myogenin and MyoD siRNAs in transient transfection, followed by 2 days of U0126 treatment. Figure 7A shows that MyoD and myogenin siRNA efficiently abrogated basal and U0126-induced protein expression, when used either alone or in combination, and also abrogated each other's protein expression (Fig. 7A, see MyoD in myogenin siRNA and vice versa). U0126-mediated p21^WAF1 ^expression was prevented in myogenin and MyoD siRNAs, as well as in combined myogenin and MyoD siRNAs, whereas it was unaffected in control siRNA-transfected cells (Fig. 7A). We then performed a transient co-transfection experiment with myogenin- and MyoD-expressing vectors, each with the puromycin resistance-expressing vector, to select the transfected cells, which were then analysed for p21^WAF1 ^accumulation. Figure 7B shows that, despite the high expression of ectopic proteins, no accumulation of p21^WAF1 ^was detected, suggesting that the increased level of myogenic transcription factors alone does not induce p21^WAF1 ^expression. The failure of ectopic myogenin and MyoD to increase p21^WAF1 ^expression might be due to inhibition of the myogenin and MyoD transactivating function, or to an epigenetic modification of the p21^WAF1 ^promoter, such as methylation, which frequently occurs in tumor cells [35]. Luciferase assay from transiently co-transfected cells with myogenin, MyoD or empty expression vectors with a p21^WAF1 ^promoter-luciferase vector (DM-Luc) showed an increased transactivating function of both myogenin and MyoD when compared with the empty vector (CMV) (Fig. 7C).

Taken together, these results suggest that, in RD cells, enhanced myogenin or MyoD alone are able to at least transactivate an ectopic p21^WAF1 ^promoter, and that MEK/ERK inhibition is required to relieve the inhibitory pathway so as to fully restore the transactivating function of endogenous myogenin and MyoD on the p21^WAF1 ^promoter.

### p21^WAF1 ^accumulation, though not myogenic differentiation, is a common feature of growth arrest in embryonal and alveolar rhabdomyosarcoma tumor-derived cell lines

**Figure 8:**
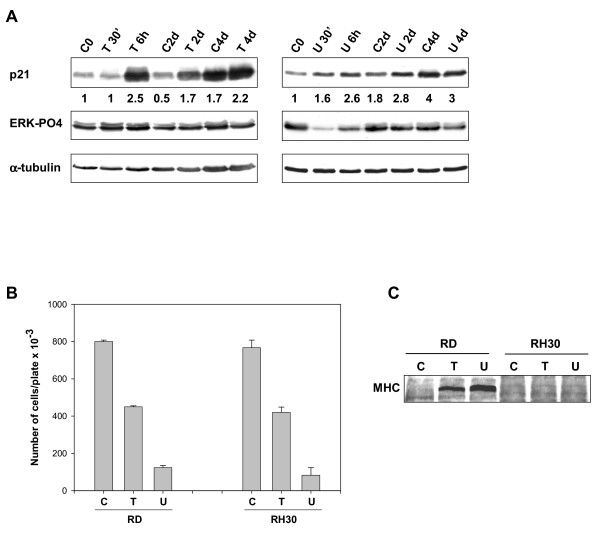
**Induction of p21^WAF1 ^expression and growth arrest in RH30 cells treated with TPA and U0126. **(A) RH30 cells treated with 10 ^-7 ^M TPA (T) or 10 μM U0126 (U) for the times indicated. Whole cell lysates were analysed by immunoblotting with a specific antibody for p21^WAF1^, phosphorylated ERK1/2 and α-tubulin expression. (B) Growth graph of RH30 and RD cells after 4 days of TPA (T) or U0126 (U) treatments. (C) Immunoblot of total lysates using specific antibody capable of recognising the sarcomeric myosin heavy chain (MHC). Similar results were obtained in two experiments.

In order to verify whether the p21^WAF1 ^expression and growth arrest induced by the TPA and MEK inhibitor U0126 are exclusive of the embryonal rhabdomyosarcoma RD cell line, we also investigated the effects of both these drugs on the alveolar rhabdomyosarcoma line RH30. Figure 8 shows that after treating cells with TPA, p21^WAF1 ^expression was significantly induced from 6 hours up to 4 days, though to a lesser extent at 4 days because there was a significant p21^WAF1 ^increase in untreated control cells. U0126 treatment also enhanced p21^WAF1 ^expression for the first 2 days, there being no increased level of p21^WAF1 ^thereafter (4 days) if compared with the untreated control cells. Transient ERK pathway activation by TPA and down-regulation by U0126 were also detected. Similarly to RD cells, TPA and U0126 both induced growth arrest of RH30 (Fig. 8B).

These data indicate that p21^WAF1^-enhanced expression is a common feature of the growth inhibitory mechanism induced by TPA and U0126 in the RH30 and RD cell lines. Since both TPA and U0126 induce myogenic differentiation markers in RD cells, we tested the expression of the myosin heavy chain (MHC) in RH30 cells. As shown in Figure 8C, unlike RD cells, neither TPA nor U0126 induced MHC expression. These preliminary data on RH30 cells suggest that TPA and U0126 fail to induce the myogenic program in spite of growth arrest.

### Forced expression of p21^WAF1 ^induces G1 arrest and reversion of anchorage-dependent growth of RD cells

**Figure 9:**
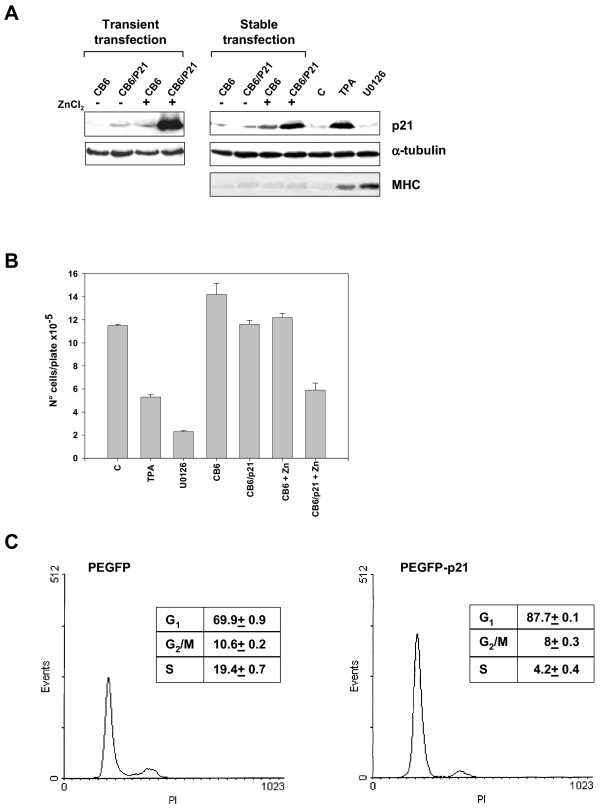
**Enforced p21^WAF1 ^expression induces growth arrest in RD cells. **(A) Immunoblotting using specific antibody capable of recognising the p21^WAF1 ^of total lysates from RD cells transfected with CB6/p21 or the empty vector (CB6), cultured without (-) or with 120 μM ZnCl_2 _(+) for 3 days before (Transient transfection) and after (Stable transfection) neomycin-selection. (B) Growth graph of same neomycin-selected RD cells cultured as in A. As a control, untransfected RD cells were left untreated (C) or treated with TPA or U0126. (C) Cell cycle distribution of RD cells transfected with empty vector PEGFP (*left*) or with PEGFP/p21 (*right*). Cell cycle distribution of GFP-expressing cells was evaluated by flow cytometry. The data shown in the insert tables are the mean ± s.e.m. of triplicates of a representative experiment. Similar results were obtained in two experiments.

**Figure 10:**
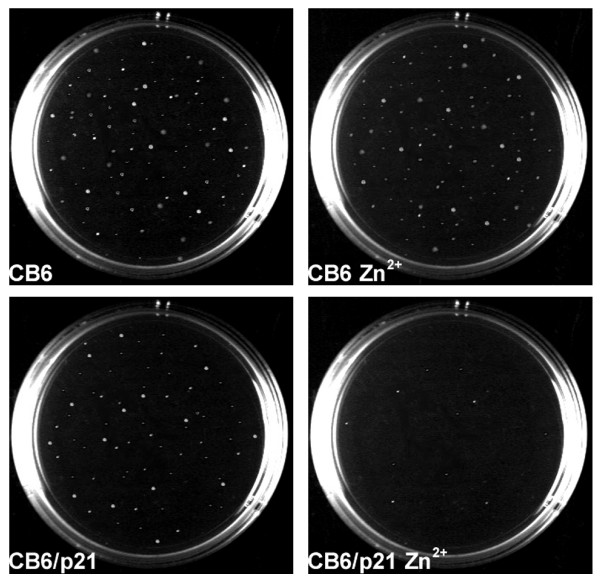
**Enforced p21^WAF1 ^expression induces reversion of anchorage independent growth. **Neomycin-selected RD cells transfected with CB6/p21 or the empty vector (CB6) were suspended in 0.33% Difco agar in the absence (CB6, CB6/p21) or presence (CB6 Zn^2+^, CB6/p21 Zn^2+^) of 120 μM ZnCl_2 _and overlaid on an 0.5% agar layer. Colonies were photographed after 14 days. Similar results were obtained in two experiments.

The main role of p21^WAF1 ^is to inhibit growth in normal and transformed cells. In order to assess the effects on cell growth of over-expression of p21^WAF1 ^in the absence of other physiological disruptions, we transfected RD cells either with vectors expressing p21^WAF1 ^(CB6-p21) under the control of the Zn+ inducible promoter [36] or with the empty vector (CB6), subsequently selecting the transfected cells with neomycin. p21^WAF1 ^was strongly expressed in a transiently transfected polyclonal population and still over-expressed in a stably transfected polyclonal population of cells under ZnCl_2 _stimulation (Fig. 9A). p21^WAF1 ^expression in stably transfected cells is comparable to that in untransfected TPA-treated cells, while no p21^WAF1 ^accumulation was observed in the empty vector. The growth potential of the p21^WAF1^-expressing cells was assessed by culturing the two polyclonal populations (CB6 and CB6/p21) for 3 days in the presence and in the absence of 120μM ZnCl_2_, and comparing them with control, TPA- and U0126-treated untransfected cells. Figure 9B shows a representative experiment of growth analysis, demonstrating 52% growth inhibition in p21^WAF1^-expressing cells, if compared with the empty vector-expressing cells, in the presence of ZnCl_2_. In addition, 53% and 80% inhibition was observed respectively in TPA- and U0126-treated cells (Fig. 9B). We also performed a FACS analysis using RD cells transfected with a vector expressing p21^WAF1^-GFP fusion protein (PEGFP-p21^WAF1^) and with a vector devoid of p21^WAF1 ^(PEGFP). The use of GFP-p21^WAF1 ^transfected cells permits cell cycle analysis in GFP-fluorescent transfected cells alone. The results of the FACS analysis demonstrate that after 48 hours of p21^WAF1 ^over-expression, DNA replication had ceased (4.6 fold reduction in cells in S-phase) and cells were arrested primarily in G1 (87.7% in G0/G1, Fig. 9C). We then investigated whether the reduced growth potential of p21^WAF1^-expressing RD cells is accompanied by reduced anchorage-independent growth, as has been demonstrated in the astrocytoma cell line [37]. We performed a soft agar clonogenic assay using stably CB6- and CB6-p21-transfected RD cells in the presence and absence of 120μM ZnCl_2_. The results, shown in Figure 10, demonstrate that RD cells expressing the empty vector (CB6) grew in the agar, forming several colonies not affected by ZnCl_2 _treatment. ZnCl_2_-mediated p21^WAF1 ^expression dramatically reduced colony formation (CB6/p21 Zn^2+^), whereas the absence of ZnCl_2 _stimulation did not (CB6/p21) (Fig. 10).

## Discussion

### ERK pathway activation or inhibition induce p21^WAF1 ^expression post-transcriptionally or transcriptionally

During the myogenic process of cultured cell lines, p21^WAF1 ^expression is controlled by myogenic transcription factors such as MyoD [10,11]. In ERMS-derived RD cells with transcriptional inactive mutated p53, the myogenic transcription factors, MyoD and myogenin, are, despite being expressed, inactive [23,27]. Inactivation of p53 and myogenic transcription factors might explain the low level of p21^WAF1 ^expression. In this paper, we have addressed the issue of how ERK pathway activation or inhibition induce growth arrest and expression of myogenic-specific genes. We show that p21^WAF1 ^accumulation is a convergence point of growth arrest signals induced by the activation or inhibition of ERKs. Nevertheless, p21^WAF1 ^accumulation varies in its extent and length of expression, it being strong and sustained after ERK activation (TPA) but transient after MEK/ERK inhibition (U0126). It is noteworthy that in U0126-treated cells, CKI inhibitor p27 expression increases concomitantly with p21^WAF1 ^and is sustained during treatment. Interestingly, when p21^WAF1 ^expression drops, p27 peaks and cyclin D1 drops as well. As a result of p21^WAF1^-mediated inhibition of the growth pathway, the hypo-phosphorylated/active form of pRb is expressed early (12 hrs) and predominantly in U0126-treated cells, and later (2 days) in TPA-treated cells.

The concomitant increase in cyclin D1 in TPA-treated cells and its decrease in U0126-treated cells may explain the stronger growth arrest response after U0126 treatment. Nevertheless, TPA-mediated withdrawal from the cell cycle may be supported by decreased cyclin A and B expression. This cell cycle expression pattern fails in untreated control cells, though the level of p21^WAF1 ^may increase as a result of culture conditions, i.e. cell confluence. Lastly, since p27 and p21^WAF1 ^may act as assembly factors [4], it is possible that the early exit from the cell cycle in U0126-treated cells is due to a combined action of p21^WAF1 ^and p27, the sustained G1 arrest then being ensured by p27 expression and by cyclin D1 loss. Regulation of p27 expression is reported to be dependent on transcriptional, post-transcriptional or protein stability mechanisms [38,39]. Nevertheless, although unable to discuss the precise mechanism of p27 increased expression by MEK inhibitor, on the basis of the above discussed results we hypothesize an involvement of p27 in growth arrest of RD cells, as it has recently been demonstrated in pancreatic cancer cells treated with MEK inhibitor U0126 [40].

As reported in the literature, p21^WAF1^ expression is mainly a result of ERK activation in a number of cell types [8,9], though it may also be due to ERK inhibition, as occurs in MCF7 cells [41]. We hypothesise that prolonged ERK activation plays an important role in supporting long-lasting p21^WAF1 ^expression in RD cells on the basis of the following results: i) U0126 prolonged treatment reduces TPA-mediated p21^WAF1 ^expression; ii) enforced expression of constitutively active MEK1 or MEK2 induces both increased p21^WAF1 ^expression and ERK pathway activation; iii) the depletion of ERK1 and ERK2 by siRNA, besides abrogating ERK1/2 expression, prevents TPA-mediated p21^WAF1 ^expression. Overall, these data prove that activation of ERKs mediates sustained p21^WAF1 ^expression. Nevertheless, while investigating the mechanisms controlling p21^WAF1^ expression, we found that TPA induces p21^WAF1 ^mRNA stabilization, which is fully responsible for p21^WAF1 ^accumulation, whereas U0126 induces p21^WAF1 ^-increased expression solely through a transcriptional mechanism. The post-transcriptional mechanism of p21^WAF1 ^induction after TPA treatment is in keeping with previous studies in the literature reporting PKC-mediated p21^WAF1^ mRNA stabilization [7].

Notably, both TPA and U0126 induce p21^WAF1 ^expression in the RH30 alveolar rhabdomyosarcoma cell line, with concomitant growth arrest. Although further investigation of the molecular mechanisms in the alveolar cell line is required, our findings suggest that p21^WAF1 ^is involved in the early growth arrest. Indeed, ERK inhibition by U0126 or activation by TPA occur in the early stages of treatments, not in the later stages. We may hypothesize that in U0126-treated RH30 cells the active ERK pathway can be restored without altering cell responsiveness to the growth-arresting signal. We are currently investigating whether these transient effects on the ERK pathway imply the involvement of other kinase pathways.

Growth arrest of RD cells has previously been studied by one group that reported an increase in the expression of p27 and p21^WAF1 ^without induction of growth arrest due to high levels of cyclins, CDKs and phospho-Rb, and by another group that reported a role of butyrate-induced p21^WAF1 ^and p27 in RD and RH30 cell line growth arrest [26,42]. Under our conditions, TPA and the MEK inhibitor disrupt a growth-signalling pathway, by affecting the MAPK cascade, and drive the cells to growth arrest and, in RD cells, myogenic differentiation (see below). This is of particular interest in light of the possibility of reversing the transformed phenotype through mechanisms, which modulate the MEK/ERK pathway.

### p38 and the ERK pathways do not cooperate in growth arrest

The apparently contrasting result regarding the activation or inhibition of the MEK/ERK pathway, both as a cause of growth arrest and myogenic differentiation, might reflect the involvement of other MAPK pathways, MAPK-p38 being the most likely candidate. Indeed, cooperation between ERK and p38 pathways in p21^WAF1^-dependent G1 cell cycle arrest has recently been reported [20]. On the other hand, the effects of ERK and p38 are reported to be dependent, respectively, on the high ERK/p38 ratio in tumor growth and on the high p38/ERK ratio in tumor arrest [18].

For these reasons, we investigated the role of the p38 pathway in p21^WAF1 ^accumulation, using the SB203580 p38 inhibitor during treatment by TPA and U0126, both previously shown by us to induce phospho/active p38 [30]. We found that the transcriptional, but not post-transcriptional mechanism of p21^WAF1 ^expression is regulated by the p38 pathway. A significant role of p38 both in growth arrest and in myogenic differentiation has recently been reported [43,44] in normal and pathological myogenic lines expressing the ectopic upstream kinase of p38. However, our results are in agreement with these data, p38 inhibition being inhibitory on U0126-mediated transcriptional mechanism of p21^WAF1 ^and myogenic transcription factors expression induced by both TPA and U0126, but is not effective on p21^WAF1 ^expression induced by TPA. As a consequence of p38 inhibition, the levels of the hypo-phosphorylated/active form of pRb in SB203580-treated cells are affected only after prolonged treatments with U0126. Conversely, neither the pRb phosphorylation status nor p21^WAF1 ^accumulation by TPA are impaired by the p38 inhibitor. It is noteworthy that the ERK/p38 ratio is predictive of growth status in a number of tumor cells [18], which suggests that, on the basis of our previous investigation [30], U0126-mediated ERK down-regulation and the sustained increase in phospho-active p38 favours persistent growth suppression.

### Myogenic transcription factors and muscle specific genes in embryonal and alveolar rhabdomyosarcoma

Both the MEK-ERK inhibitor and TPA induce myogenic-specific gene expression, with MHC accumulation in U0126-treated cells occurring earlier than in TPA-treated cells. Early myogenin accumulation followed by MyoD shows that the myogenic program is rapidly rescued in ERK-depleted cells.

Cyclin D1 might also be responsible for the delay in the activation of myogenic transcription factors [45] in TPA-treated cells; by contrast, cyclin D1 is down-regulated by U0126 alone or together with TPA, leading to a rapid start of the myogenic program. Remarkably, myogenin and MyoD expression, strongly induced by U0126 in both the presence and absence of TPA, are down-regulated by the p38 inhibitor, thereby paralleling the pattern observed in p21^WAF1 ^expression. In view of these results, we hypothesize that MyoD, as previously shown in normal myogenesis [10,11], and even myogenin might transactivate p21^WAF1 ^expression in MEK inhibitor-treated cells. Indeed, U0126-mediated p21^WAF1 ^expression requires myogenin and MyoD, as demonstrated by its drastic inhibition in myogenin and MyoD siRNA experiments. However, MyoD- or myogenin-forced expression in RD cells, while inducing an ectopic p21^WAF1 ^promoter, does not induce an increase in the p21^WAF1 ^level. The discrepancy between the inability of forced myogenin and MyoD expression to induce p21^WAF1 ^and the ability of these two transcription factors to transactivate an ectopic promoter, in transfected RD cells, suggests that inhibitory pathways responsible for p21^WAF1 ^repression operate at the level of the p21^WAF1 ^endogenous promoter. It is noteworthy that the authors of another study [43] did not detect p21^WAF1 ^promoter transactivation by ectopic MyoD in RD cells. However, this discrepancy may depend on differences in the experimental approach used as the authors of that study addressed the issue of whether the upstream p38 kinase, namely MKK6E, synergistically affects MyoD transactivating function. We are mainly interested in clarifying whether the rescue of myogenic transcription factors expression and functions might be responsible for the restored p21^WAF1 ^transcription. Our results specifically concerning the positive role of the p38 pathway in p21^WAF1 ^transcription are, however, in agreement with those reported in the aforementioned study. Indeed, p38 inhibitor was found to drastically inhibit the myogenic transcription factor as well as p21^WAF1 ^and sarcomeric myosin expression. Thus, it is possible that MEK/ERK inhibition, following U0126 treatment, leads to p21^WAF1 ^transcription by unmasking of the transcriptional site targets of MyoD and myogenin, on the one hand, and directs RD cells towards growth arrest and the differentiation program by enhancing myogenic transcription factors levels, on the other. Unlike RD cells, RH30 cells do not undergo myogenic differentiation despite being induced to growth arrest.

### Ectopic p21^WAF1 ^induces growth arrest and reversal of the onco-phenotype independently of the ERK pathway

The role of p21^WAF1 ^in RD cell growth arrest is demonstrated here by the growth inhibition (53%) induced by forced expression of the p21^WAF1 ^inducible vector and by the FACS analysis of RD cells transfected with p21^WAF1^-GFP.

These results, together with our previous data on early G1 arrest in ERK pathway-depleted cells [30], suggest that p21^WAF1 ^and the rescue of myogenic transcription factor functions play a role in dismantling the proliferative incentive, thereby rapidly driving the cells to G1 arrest.

In view of these results, combined with the body of evidence showing that p21^WAF1 ^functions as a tumor suppressor, we tested focus formation in soft agar of p21^WAF1 ^stably transfected RD cells, revealing a dramatic loss of anchorage independent growth. This result demonstrates that p21^WAF1 ^is, by itself, able to override the transforming potential of RD cells. These data, though promising with regard to the role of p21^WAF1 ^alone in reverting malignant growth, warrant further research on the anchorage independent growth pathways that may be affected by high p21^WAF1 ^levels.

## Conclusion

In this study we highlight the importance of targeting the MEK/ERK pathway as a means of restoring the expression of the tumor suppressor p21^WAF1 ^as well as the growth arrest mechanism. The results of this study suggest that the targeting of ERKs to rescue p21^WAF1 ^expression and myogenic transcription factor functions leads to the reversal of the Rhabdomyosarcoma phenotype. The inhibition of the MEK/ERK pathway might, therefore, prove to be a novel therapeutic approach for the reversal of the Rhabdomyosarcoma phenotype.

## Methods

### Cell cultures and treatments

The human embryonal RD (ATCC, Rockville MD) and alveolar RH30 (DSMZ, Braunschweig, Germany) rhabdomyosarcoma cells were cultured respectively in Dulbecco's modified Eagle's and RPMI medium containing 10% fetal calf serum (Hyclone, Logan UT) supplemented with glutamine and gentamycin (GIBCO-BRL Gaithersburg, MD). One day after plating, cells were treated with 10^-7 ^M TPA (Sigma, St. Louis, MO) or with 10 μM kinase inhibitors U0126 (Promega, Madison, WI) and/or 5 μM SB203580, or SB202474 as a negative control (Calbiochem, La Jolla CA), for the times shown in the figures. Actinomycin D (0.05 μg/ml) was incubated for 1 hr before stimulation with TPA or U0126, in complete medium; cycloheximide (10μM) (Sigma, St. Louis, MO) was incubated after 1 hr of TPA treatment in complete medium.

### Immunoblot analysis

Cells were lysed in 2% SDS containing 2 mM phenyl-methyl sulphonyl fluoride (PMSF) (Sigma), 10 μg/ml antipain, leupeptin and trypsin inhibitor, 10 mM sodium fluoride and 1 mM sodium orthovanadate (all from Sigma) and sonicated for 30 sec. Proteins of whole cell lysates were assessed using the Lowry method [46], and equal amounts were separated on SDS-PAGE. The proteins were transferred to a nitrocellulose membrane (Schleicher & Schuell, BioScience GmbH, Germany) by electroblotting. The balance of total protein levels was confirmed by staining the membranes with Ponceau S (Sigma). Immunoblottings were performed with the following antibodies: anti-p21 (C-19), anti-cyclin D1 (M-20) and D3 (C-16), E (HE12), A (H-432) and B (H-20) cyclins, CDK2 (M2) and 4 (H-22), -pRb (C-15), anti-myogenin (F-D5), anti-ERK2 (C-14, positive also for ERK1) anti-phospho-ERKs (E-4) and α-tubulin (B-7) (all from Santa Cruz Biotechnology, Santa Cruz CA), MyoD (clone 5.8A, Novocastra Newcastle, UK, or C-20 from Santa Cruz Biotechnology) and anti-MHC (MF20, gift from Fichman D). Peroxidase-conjugate anti-mouse or anti-rabbit IgG (Amersham-Pharmacia Biotech, UK or Santa Cruz) were used for enhanced chemiluminescence (ECL) detection.

### Northern blot analysis

Cells were collected and lysed in Trizol reagent (GIBCO-BRL). Total RNA was isolated according to the manufacturer's instructions. 10 μg of total RNA was resolved on a formaldehyde/agarose gel, and transferred to GeneScreen Plus (DuPont, Bad Homburg, Germany) membranes. Filters were cross-linked by baking at 80°C for 2 hrs, then hybridised overnight with 1 × 10^6 ^to 2 × 10^6 ^cpm of ^32^P labelled DNA probes per ml. DNA probes were labelled by random priming to a specific activity of approximately 0.5 × 10^9 ^cpm/μg. The membranes were washed at a final stringency of 0.1 × SSC, 0.5% SDS at 60°C. The p21^WAF1^, MyoD and myogenin probes was obtained from the plasmid described below, while cyclin D1 probe was kindly provided by Dr. A. Arnold [47] and GAPDH vector was provided by ATCC.

### Plasmids and transfections

One day after plating, RD cells were transfected with all the plasmids using Lipofectamine Plus reagent (Invitrogen, Italy) according to the manufacturer's instructions (GIBCO-BRL, Gaithersburg, MD). RNA interference experiments were performed with siRNA for ERK1 and ERK2, myogenin and/or MyoD (Sancta Cruz Biotechnology) using Lipofectamine 2000 reagent (Invitrogen, Italy), according to the manufacturer's instructions. Briefly, cells were plated at 40–50% confluence and transfected after 24 hr with 100 nM siRNA, which we ascertained was sufficient to detect maximum fluorescence using fluorescein-conjugated control siRNA. For the luciferase assay, the human p21^WAF1 ^promoter construct DM-Luc (gift from Dr. P. Dotto) was co-tranfected into RD cells together with CMV β-Galactosidase expressing vector as the internal standard to control for transfection efficiency. One day after transfection, cells were treated with TPA or left untreated for 24 hrs. Total lysates were processed for luciferase activity according to the manufacturer's instructions (Promega Italia). Luciferase activity was normalized for the expression level of transfected β-Galactosidase protein [48]. Alternatively, DM-Luc was co-transfected with plasmid expressing MyoD [49] or myogenin [50]; after 48 hrs, cells were harvested, lysed and processed for luciferase activity as described above. For p21^WAF1 ^expression analysis, RD cells were also transiently transfected with: MyoD or myogenin together with puromycin resistance-expressing vectors (pPur, Clontech laboratories Gmbh, Heidelberg, Germany) to select transfected cells with puromycin (4μg/ml); with constitutively active MEK1 or MEK2 kindly provided by N. Ahn [51]. After 48 hours, cells were harvested, lysed and processed for immunoblotting. RD cells stably expressing p21^WAF1 ^or the empty vector were prepared by transfecting cells with a plasmid encoding full length human p21^WAF1^, aa 1–164 (Zinc-inducible vector pMT-CB6/p21) and the empty vector (Zinc-inducible pMT-CB6) carrying neomycin resistance, donated by Asada M [36], followed by selection in G418 (0.5 mg/ml) for 3 weeks. G418-resistant clones were pooled for a representative stock of stably transfected cells and re-plated for stimulation with 120 μM ZnCl_2 _for 3 days. Cells were processed both for p21^WAF1 ^expression analysis and for the number of cells counted in the hemocytometer chamber.

For transient expression of both pEGFP-p21^WAF1 ^full length and pEGFP (gift from Asada M.), RD cells were transfected and collected for FACS analysis 24 hrs later.

### FACS analysis

Cells were harvested by trypsin-EDTA and washed; pellets were then resuspended in PBS additioned with 1% paraformaldehyde (final concentration of 0.5%) left at 4°C for 1 hr. The fixed cells were then washed with PBS twice, resuspended in 0.3 ml of 50% FCS in PBS, additioned with 0.9 ml of 70% ethanol and left at 4°C in the dark for no longer than 2 days before FACS analysis (Coulter Epics XL Flow Cytometer, Beckman Coulter Ca, USA).

### Colony-forming assays in semisolid agar

Colony-forming assays were based on standard methods. Briefly, 2 × 10^4 ^ cells were resuspended in 4 ml of 0.33% special Noble agar (Difco, Detroit, MI) and plated (6 cm plate) in growth medium containing 0.5% soft agar. Colonies were photographed 14 days after plating.

## List of abbreviations

RD, rhabdomyosarcoma cell line; MEK, Mitogen-activated protein Extracellular Kinase; ERK, Extracellular signal-Regulated protein Kinase.

## Authors' contributions

CC and FM contributed equally to the acquisition of most of the data presented. AS contributed to the beginning of the research, and in particular to the p21-promoter luciferase assay. AM and CG contributed to the first analysis of p21^WAF1 ^expression by Western blot of the MEK inhibitor- and TPA-treated cells. PDC performed the FACS analysis. BMZ, conceived the study and wrote the manuscript.

## Supplementary Material

Additional File 1 **TPA-mediated ERK-pathway activation and U0126-mediated ERK-pathway down-regulation. **RD cells were treated with 10^-7 ^M TPA and/or 10 μM U0126 (U) for the times indicated. Whole cell lysates from untreated (C) or TPA- (TPA) and U0126-treated (U0126) cells were separated on SDS-PAGE and analysed by immunoblotting with antibody specific for phospho-ERKs. Click here for file

Additional File 2 **Expression of unaltered cell cycle markers during growth arrest induced by TPA. **RD cells were treated with 10^-7 ^M TPA for the times indicated. Whole cell lysates from untreated (C) or TPA-treated cells (TPA) were separated on SDS-PAGE and analysed by immunoblotting with specific antibodies for the proteins indicated. The data shown are representative of three independent experiments. Click here for file

Additional File 3 **p21^WAF1 ^expression after transcription or translation inhibition. **(A) Immunoblotting of total lysates from RD cells left untreated (C) or treated with U0126 or TPA for 5 hours with or without a 1 hr 0.05 μg/ml of actinomycin D (ActD) pre-treatment. (B) Immunoblotting of total lysates from RD cells left untreated (C) or pre-treated for 1 hr with TPA and then treated with 10 μM cycloheximide (TPA+CHX) for indicated times, TPA and cycloheximide were also added alone (TPA, CHX) for the indicated times. Immunoblots were performed using a specific antibody capable of recognising p21^WAF1^. α-tubulin expression levels show equal loading. Click here for file

Additional File 4 **Effects of p38 inhibition on cyclin D1 expression. **RD cells were left untreated (C), treated with TPA or U0126 (U) or pre-treated with 5 μM SB 203580 for 1 hour and then left untreated (SB) or treated with TPA (SB+TPA) or U0126 (SB+U) for the times indicated. Immunoblots of total lysates were performed using specific antibody capable of recognising cyclin D1. Click here for file

Additional File 5 **Effects of p38 inhibitor SB 203580 and its inactive analogue SB 202474 on myogenin expression. **RD cells were pre-treated for 1 hour or 4 days with SB 203580 or SB 202474 (5 μM of both) and then treated with TPA for 6 hours or 4 days. Immunoblots of total lysates were performed using specific antibody capable of recognising myogenin. α-tubulin expression levels show equal loading. Click here for file
