# Supplementary figures and images for: p21WAF1 expression induced by MEK/ERK pathway activation or inhibition correlates with growth arrest, myogenic differentiation and onco-phenotype reversal in rhabdomyosarcoma cells
Source: Mol Cancer. 2005 Dec 13;4:41. doi: 10.1186/1476-4598-4-41 (PMC1343585; doi:10.1186/1476-4598-4-41)

C

TPA

U

U+TPA

3h

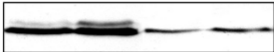

12h

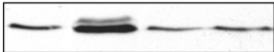

2d

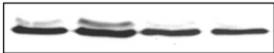

4d

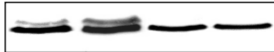

ERK-PO<sub>4</sub>

Supplement: Additional File 1 — TPA-mediated ERK-pathway activation and U0126-mediated ERK-pathway down-regulation. RD cells were treated with 10-7 M TPA and/or 10 μM U0126 (U) for the times indicated. Whole cell lysates from untreated (C) or TPA- (TPA) and U0126-treated (U0126) cells were separated on SDS-PAGE and analysed by immunoblotting with antibody specific for phospho-ERKs. [file 1476-4598-4-41-S1.pdf]

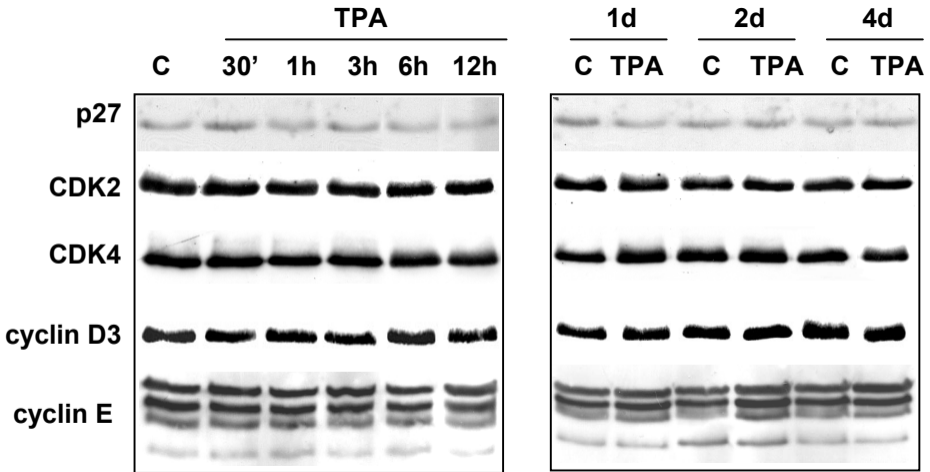

Supplement: Additional File 2 — Expression of unaltered cell cycle markers during growth arrest induced by TPA. RD cells were treated with 10-7 M TPA for the times indicated. Whole cell lysates from untreated (C) or TPA-treated cells (TPA) were separated on SDS-PAGE and analysed by immunoblotting with specific antibodies for the proteins indicated. The data shown are representative of three independent experiments. [file 1476-4598-4-41-S2.pdf]

**A**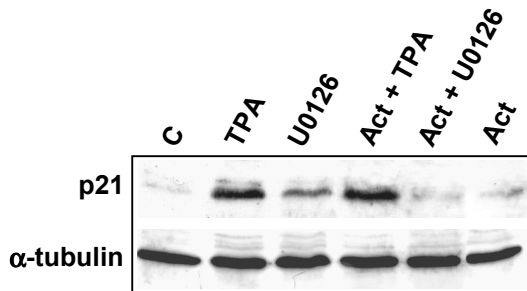**B**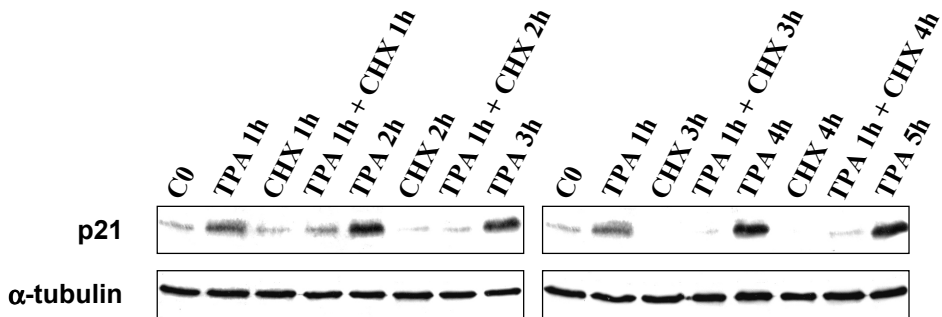

Supplement: Additional File 3 — p21WAF1 expression after transcription or translation inhibition. (A) Immunoblotting of total lysates from RD cells left untreated (C) or treated with U0126 or TPA for 5 hours with or without a 1 hr 0.05 μg/ml of actinomycin D (ActD) pre-treatment. (B) Immunoblotting of total lysates from RD cells left untreated (C) or pre-treated for 1 hr with TPA and then treated with 10 μM cycloheximide (TPA+CHX) for indicated times, TPA and cycloheximide were also added alone (TPA, CHX) for the indicated times. Immunoblots were performed using a specific antibody capable of recognising p21WAF1. α-tubulin expression levels show equal loading. [file 1476-4598-4-41-S3.pdf]

12h

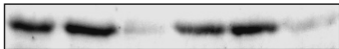

2d

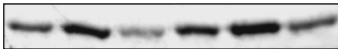

5d

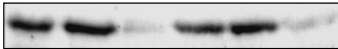

**Cyclin D1**

Supplement: Additional File 4 — Effects of p38 inhibition on cyclin D1 expression. RD cells were left untreated (C), treated with TPA or U0126 (U) or pre-treated with 5 μM SB 203580 for 1 hour and then left untreated (SB) or treated with TPA (SB+TPA) or U0126 (SB+U) for the times indicated. Immunoblots of total lysates were performed using specific antibody capable of recognising cyclin D1. [file 1476-4598-4-41-S4.pdf]

C 4d

TPA 4d

SB 233580 4d

SB 202474 4d

C 4d + TPA 6h

SB 233580 4d + TPA 6h

SB 202474 4d + TPA 6h

SB 233580 1h + TPA 4d

SB 202474 1h + TPA 4d

Myogenin

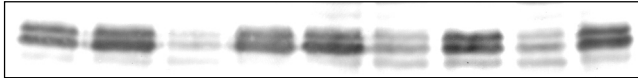

$\alpha$ -tubulin

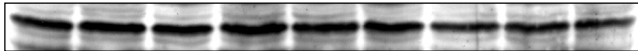

Supplement: Additional File 5 — Effects of p38 inhibitor SB 203580 and its inactive analogue SB 202474 on myogenin expression. RD cells were pre-treated for 1 hour or 4 days with SB 203580 or SB 202474 (5 μM of both) and then treated with TPA for 6 hours or 4 days. Immunoblots of total lysates were performed using specific antibody capable of recognising myogenin. α-tubulin expression levels show equal loading. [file 1476-4598-4-41-S5.pdf]
